# Supplementary material for: HIV-1 infection regulates gene expression by altering alternative polyadenylation correlated with CPSF6 and CPSF5 redistribution
Source: mBio. 2025 Dec 17;17(1):e02865-25. doi: 10.1128/mbio.02865-25 (PMC12802250; doi:10.1128/mbio.02865-25)
Supplement: Supplemental Figures Part 1 — Figures S1 and S2. [file mbio.02865-25-s0001.docx]

**SUPPLEMENTARY FIGURES**

**
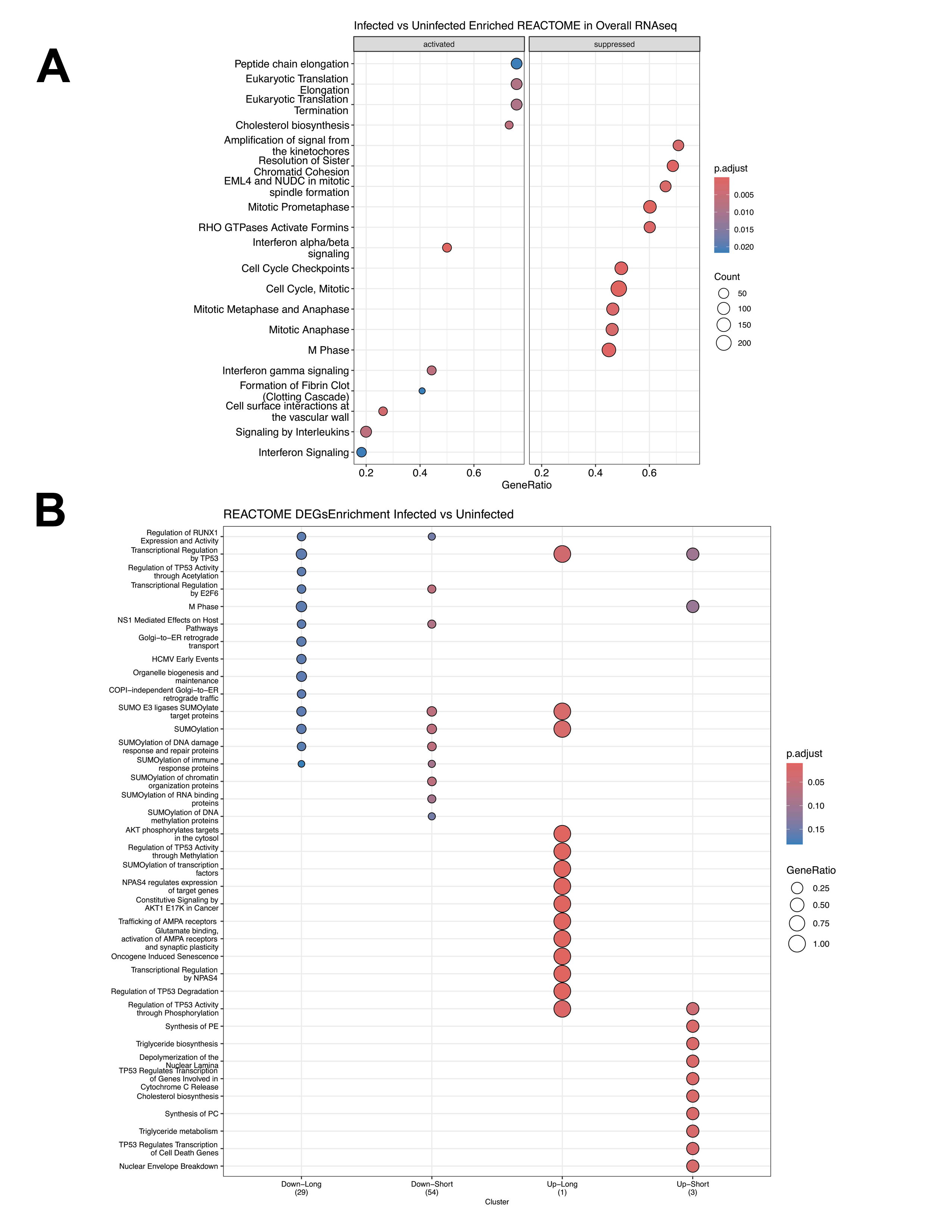
**

A

B

**
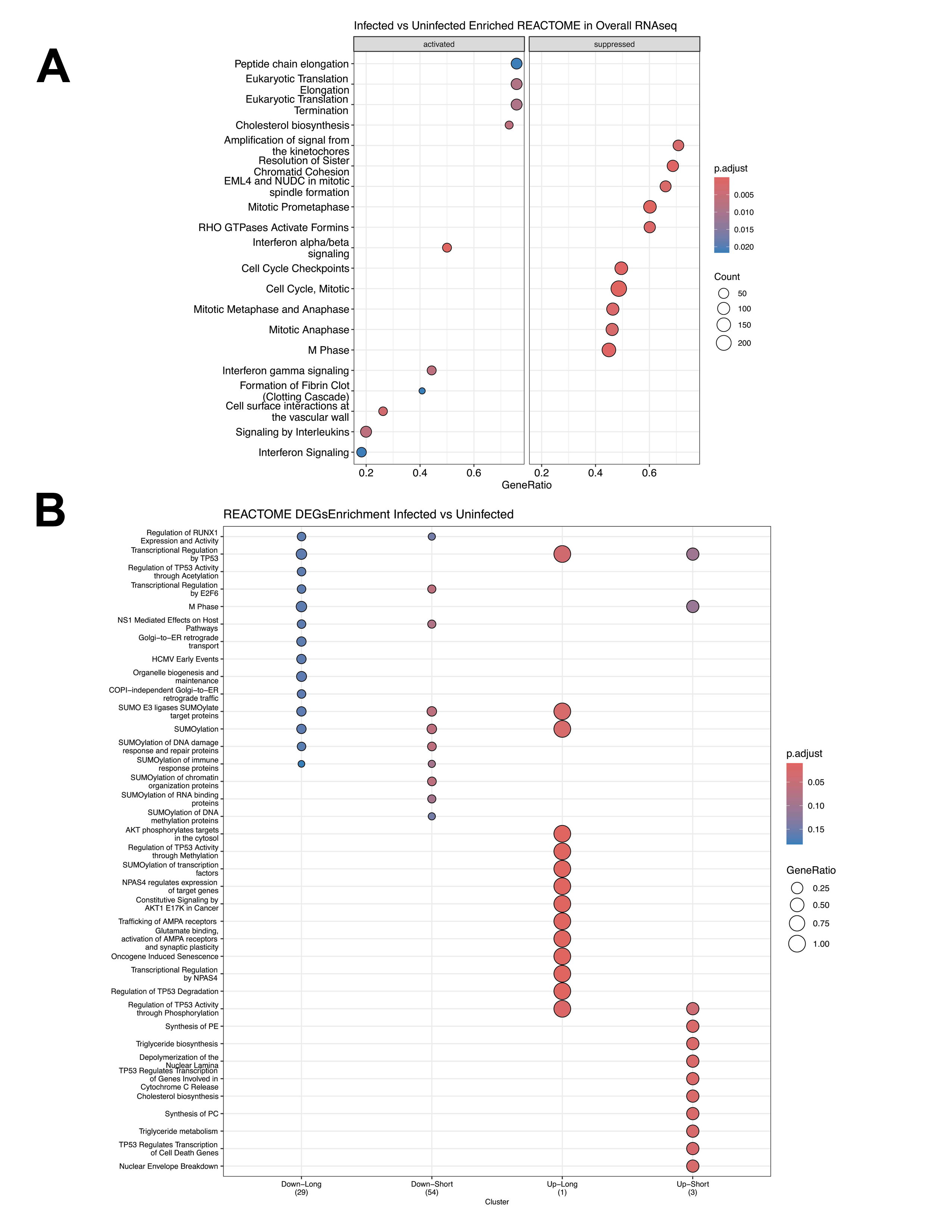
**

**Figure S1.** **REACTOME pathway analysis from RNA-seq in A549 cells, related to Figure 1.** **(A-B)** Human A549 cells were challenged with HIV-1-GFP viruses at an MOI of ~2 for 24 hours. Total RNA from three infected and three mock-infected samples was sequenced using RNA-seq, as described in the Methods section. **(A)** Pathway enrichment analysis of differentially expressed genes (DEGs). Dot plot shows top 20 enriched REACTOME pathways of the genes that were significantly changed by HIV-1 infection. The size of the dot is based on gene count enriched in the pathway, and the color of the dot shows the pathway enrichment significance (p-value). **(B)** Pathway enrichment analysis of DEGs clustered according to 3’UTR lengths (down-long; down-short; up-long; up-down). The size of the dot is based on GeneRatio enriched in the pathway, and the color of the dot shows the pathway enrichment significance.

**Figure S2.** **Gene expression analysis from PAC-seq in A549 cells, related to Figure 2.** Human A549 cells were challenged with HIV-1-GFP **(A)**, HIV-1-N74D **(B)** or HIV-1-A77V **(C)** viruses at an MOI of ~2 for 48 hours**.** Total RNA from two infected and two mock-infected samples was sequenced to analyze changes in gene expression, as described in the Methods section. **(A-C)** Volcano plot shows differentially expressed genes in PAC-Seq data. The X-axis represents differences in gene expression as Log_2_-fold changes. A positive Log_2_-fold indicates upregulation (red) of the corresponding gene in infected cells, while a negative Log_2_-fold indicates downregulation (blue). The Y-axis represents the statistical significance of the results, expressed as P-value. **(D-E)** Gene ontology analysis of all genes that were upregulated **(D)** and downregulated **(E)** by HIV-1 infection. The color of the bars represents the P-adjusted value.
