# Supplementary material for: HIV-1 infection regulates gene expression by altering alternative polyadenylation correlated with CPSF6 and CPSF5 redistribution
Source: mBio. 2025 Dec 17;17(1):e02865-25. doi: 10.1128/mbio.02865-25 (PMC12802250; doi:10.1128/mbio.02865-25)
Supplement: Supplemental Figures Part 2 — Figures S3 to S7. [file mbio.02865-25-s0002.docx]

**Figure S3. HIV-1 infection induces changes in SLFN5 expression in human primary cells, related to Figure 4.** Human primary cells: **(A)** monocytes, **(B)** macrophages, and **(C)** CD4^+^ T cells were infected with HIV-1-GFP at MOI of 2 for 96 h. Cells were lysed, and proteins were analyzed by western blotting using the indicated antibodies. Virus presence was assessed using anti-p24 antibodies, and anti-GAPDH antibodies were used as a protein loading control. Each experiment was performed at least three times with different donors, and a representative image is shown. Graphs show the average densitometry quantification of two replicates with standard deviation. Infection was assessed by flow cytometry as the percentage of GFP-positive cells. Significance was determined using unpaired t-test; *p<0.05; **p<0.01; ***p<0.001.

**Figure S4. Changes in SLFN5 expression induced by HIV-1 depend on its MOI and the time of infection, related to Figure 4.** Human A549 cells were infected under the following conditions: **(A)** HIV-1-GFP at increasing MOIs for 48 h; **(B)** HIV-1-luc at an MOI of 2 for 48, 72 and 96 h. Cells were lysed, and proteins were analyzed by western blot using the indicated antibodies. The presence of virus was assessed using anti-p24 antibodies, and anti-GAPDH antibodies were used as a protein loading control. Each experiment was repeated at least three times, and a representative image is shown. Graphs show the average densitometry quantification of three replicates with standard deviation. Infection was assessed by flow cytometry as the percentage of GFP-positive cells at the indicated hours post-infection (hpi). Significance was determined using ANOVA multiple comparisons tests; *p<0.05; **p<0.01; ***p<0.001; ns, not significant.

**Figure S5. Inhibition of HIV-1 reverse transcription by nevirapine partially changes SLFN5 expression, related to Figure 4.** Human A549 cells were infected with three independent HIV-1-GFP preparations at an MOI of 2 with or without 10 µM Nevirapine (Nev) for 48h. Cells were lysed, and proteins were analyzed by western blot using the indicated antibodies. Virus presence was assessed using anti-p24 antibodies, and anti-GAPDH antibodies were used as a protein loading control. Each experiment was repeated at least three times, and a representative image is shown. Graphs show the average densitometry quantification of three replicates with standard deviation. Infection was assessed by flow cytometry as the percentage of GFP-positive cells. Significance was determined using ANOVA multiple comparisons tests; ***p<0.001; ns, not significant.

**Figure S6.** **Loss of CPSF6 expression induces changes in VMA21 protein levels in A549 cells, related Figure 5.** Human A549 WT, NT#H1, CPSF6-KO#B4, CPSF6-KO#B7, and CPSF6-KO#C8 cells were lysed, and proteins were analyzed by Western blotting using anti-VMA21 and anti-CPSF6 antibodies. As loading control, we utilized anti-GAPDH antibodies. Each experiment was repeated at least three times, and a representative experiment is shown. Graphs show the average densitometry quantification of three replicates with standard deviation. Significance was determined using ANOVA multiple comparisons tests; ***p<0.001; ns, not significant.

**Figure S7. HIV-1 infection in CPSF6-KO cells slightly increases SLFN5 expression, related Figure 6.** Human A549 WT, CPSF6-KO#B4, CPSF6-KO#B7, and CPSF6-KO#C8 cells were infected with HIV-1-GFP at an MOI of 2 for 48 h. Cells were lysed, and proteins were analyzed by western blot using the indicated antibodies. Virus presence was assessed using anti-p24 antibodies, and anti-GAPDH antibodies were used as a protein loading control. Each experiment was repeated at least three times, and a representative experiment is shown. Graphs show the average densitometry quantification of three replicates with standard deviation. Infection was assessed by flow cytometry as the percentage of GFP-positive cells. Significance was determined using unpaired t-test; ***p<0.001; ns, not significant.
