# Supplementary material for: HIV-1 infection regulates gene expression by altering alternative polyadenylation correlated with CPSF6 and CPSF5 redistribution
Source: mBio. 2025 Dec 17;17(1):e02865-25. doi: 10.1128/mbio.02865-25 (PMC12802250; doi:10.1128/mbio.02865-25)
Supplement: Captions — for Tables S1 to S3. [file mbio.02865-25-s0003.docx]

**SUPPLEMENTARY TABLES**

**Table S1.** PolyAminer analysis of PAC-Seq 3'UTR APA data from human A549 cells infected with viruses HIV-1-GFP **(A)**, HIV-1-N74D-GFP **(B)** or HIV-1-A77V-GFP **(C)** compared to A549 cells mock-infected, related to Figure 2.

**Table S2.** PolyAminer analysis of PAC-Seq 3'UTR APA data from Human primary CD4+ T cells infected with viruses HIV-1-GFP **(A)**, HIV-1-N74D-GFP **(B)** or HIV-1-A77V-GFP **(C)** compared to T cells mock-infected, related to Figure 3.

**Table S3.** PolyAminer analysis of PAC-Seq 3'UTR APA data from human A549- CPSF6- KO cells: CPSF6-KO#B4 **(A)**, CPSF6-KO#B7 **(B)**, and CPSF6-KO#C8 **(C)** compared to A549 parental cells, related to Figure 5B.
